# Disease-modifying drugs for multiple sclerosis and subsequent health service use

**DOI:** 10.1177/13524585211063403

**Published:** 2021-12-24

**Authors:** Huah Shin Ng, Feng Zhu, Elaine Kingwell, Yinshan Zhao, Shenzhen Yao, Okechukwu Ekuma, Lawrence W Svenson, Charity Evans, John D Fisk, Ruth Ann Marrie, Helen Tremlett

**Affiliations:** Department of Medicine, Division of Neurology and the Djavad Mowafaghian Centre for Brain Health, University of British Columbia, Vancouver, BC, Canada; Department of Medicine, Division of Neurology and the Djavad Mowafaghian Centre for Brain Health, University of British Columbia, Vancouver, BC, Canada; Department of Medicine, Division of Neurology and the Djavad Mowafaghian Centre for Brain Health, University of British Columbia, Vancouver, BC, Canada/Research Department of Primary Care & Population Health, University College London, London, UK; Department of Medicine, Division of Neurology and the Djavad Mowafaghian Centre for Brain Health, University of British Columbia, Vancouver, BC, Canada; College of Pharmacy and Nutrition, University of Saskatchewan, Saskatoon, SK, Canada/Health Quality Council, Saskatoon, SK, Canada; Department of Community Health Sciences, Rady Faculty of Health Sciences, University of Manitoba, Winnipeg, MB, Canada; Alberta Health, Edmonton, AB, Canada/Division of Preventive Medicine & School of Public Health, University of Alberta, Edmonton, AB, Canada/Community Health Sciences, Cumming School of Medicine, University of Calgary, Calgary, AB, Canada; College of Pharmacy and Nutrition, University of Saskatchewan, Saskatoon, SK, Canada; Nova Scotia Health Authority and the Departments of Psychiatry, Psychology and Neuroscience, and Medicine, Dalhousie University, Halifax, NS, Canada; Departments of Internal Medicine and Community Health Sciences, Max Rady College of Medicine, Rady Faculty of Health Sciences, University of Manitoba, Winnipeg, MB, Canada; Department of Medicine, Division of Neurology and the Djavad Mowafaghian Centre for Brain Health, University of British Columbia, Vancouver, BC, Canada/Division of Neurology, Department of Medicine, Faculty of Medicine, UBC Hospital, Vancouver, BC, Canada

**Keywords:** Disease-modifying drugs, health services, hospitalization, multiple sclerosis, physician services

## Abstract

**Objective:**

We assessed the relationship between the multiple sclerosis (MS) disease-modifying drugs (DMDs) and healthcare use.

**Methods:**

Persons with MS (aged ⩾18 years) were identified using linked population-based health administrative data in four Canadian provinces and were followed from the most recent of their first MS/demyelinating event or 1 January 1996 until the earliest of death, emigration, or study end (31 December 2017 or 31 March 2018). Prescription records captured DMD exposure, examined as any DMD, then by generation (first-generation (the injectables) or second-generation (orals/infusions)) and individual DMD. The associations with subsequent all-cause hospitalizations and physician visits were examined using proportional means model and negative binomial regression.

**Results:**

Of 35,894 MS cases (72% female), mean follow-up was 12.0 years, with person-years of DMD exposure for any, or any first- or second-generation DMD being 63,290, 54,605 and 8685, respectively. Any DMD or any first-generation DMD exposure (versus non-exposure) was associated with a 24% lower hazard of hospitalization (adjusted hazard ratio, aHR: 0.76; 95% confidence intervals (CIs): 0.71–0.82), rising to 29% for the second-generation DMDs (aHR: 0.71; 95% CI: 0.58–0.88). This ranged from 18% for teriflunomide (aHR: 0.82; 95% CI: 0.67–1.00) to 44% for fingolimod (aHR: 0.56; 95% CI: 0.36–0.87). In contrast, DMD exposure was generally not associated with substantial differences in physician visits.

**Conclusion:**

Findings provide real-world evidence of a beneficial relationship between DMD exposure and hospitalizations.

## Introduction

Disease-modifying drug (DMD) efficacy is typically established after short clinical trials in highly selected multiple sclerosis (MS) patients.^1^ However, in clinical practice, DMDs are used in the wider MS population. A recent study showed that nearly 20% of people with MS had some comorbidity and approximately one in six were aged ⩾50 years when first receiving a DMD.^2^ However, such individuals are typically excluded from clinical trials.^2^ Understanding the benefits of the DMDs in the broader MS populations treated in the “real-world” setting is needed for therapeutic-related decision-making. Health service use, particularly hospitalizations, represents a major burden for society, patients, and families. Population-based longitudinal studies of health care use are needed to assess the benefits of the DMDs and facilitate healthcare planning.^3^ Prior studies have not been population-based, have been limited to private health insurance enrollees^4,5^ or were ecological in design and unable to assess DMD use at the individual patient level.^6,7^

We investigated the relationship between DMD exposure and health service use in a MS population using individual patient-level linked administrative data collected over a 22-year period.

## Methods

### Data sources

This was a multi-region, population-based observational study. Multiple administrative health data-bases covering four provinces (British Columbia, Saskatchewan, Manitoba, Nova Scotia), representing nearly 25% of Canada’s population,^8^ were linked for each individual resident within each province. The administrative data included the following: physician and hospital data,^9,10^ capturing all physician visits and hospitalizations, coded using the International Classification of Diseases (ICD) system; the provincial health insurance registries,^11^ capturing demographic information (residency status, sex, birthdates, and residential postal codes, and for Manitoba/Saskatchewan, death dates); and vital statistics data,^12^ capturing death records in British Columbia/Nova Scotia. Prescription data provided all pharmacy dispensed prescriptions (including dates and number of days supplied/quantity dispensed; British Columbia,^13^ Manitoba, and Saskatchewan). In Nova Scotia, the Dalhousie MS Research Unit database provided DMD prescription start and stop dates.

### Study population

We identified MS cases using a validated algorithm (⩾3 MS diagnostic codes (ICD-9/10 340/G35) or ⩾1 dispensation of a DMD).^14^ Once identified, cases were assigned an index date, defined as the most recent of the first MS or related demyelinating disease code captured in the physician/hospital data or first MS DMD prescription filled (Supplementary Tables 1–2); an individual’s 18th birthday; or 1 January 1996 (British Columbia), 1 April 1996 (Manitoba), 1 January 1997 (Saskatchewan), or 1 January 1998 (Nova Scotia). These dates represent the first availability of prescription data (including the MS DMDs), and first full year of government financial coverage of the DMDs. One year of provincial residency pre-index date was required; all identified persons with MS were followed from their index date until the earliest of death, emigration, or study end (31 December 2017 (British Columbia, Manitoba, Nova Scotia) and 31 March 2018 (Saskatchewan) based on the last availability date).

Cohort characteristics—sex, age, calendar-year, and socioeconomic status (estimated from neighborhood-level income via postal codes)^15^—were described at the index date. Comorbidity in the year pre-index date was measured using the Charlson Comorbidity index (excluding hemiplegia and paraplegia to avoid misclassifying MS symptoms).^16^

### Exposure

First- and second-generation DMD availability over the study period is shown in Supplementary Table 2, including the year of each DMD’s approval for use in MS by Health Canada. Briefly, the first-generation DMDs included the injectables (i.e. beta-interferon and glatiramer acetate), and the second-generation included the infusions and orally administered DMDs.^17^ DMD exposure periods were determined based on the number of days supplied (British Columbia, Manitoba), or the quantity dispensed (Saskatchewan), or the DMD start/stop dates (Nova Scotia). Discontinuation was defined as no further dispensations for that DMD (lasting ⩾90 consecutive days), plus a 30-day grace period (all provinces); that is, discontinuation date = last DMD dispensed date + days supplied + 30 days.^18^ Except for alemtuzumab and ocrelizumab, whereby a person was considered exposed for a full 12- or 6-month post-dispensation, respectively, and discontinued thereafter if no further dispensations (plus the 30-day grace period).

An individual’s exposure status could change over time (i.e. time-dependent variable) and was assessed as periods of any exposure or no exposure, then by generation and individual DMD.

### Outcomes

The primary and secondary outcomes were all-cause hospitalizations (events) and number of all physician service use (visits), respectively. Hospitalizations included day surgery, but not infusions (e.g. natalizumab or alemtuzumab).^19^ To avoid double counting, any new hospitalization occurring within 24 hours (e.g. due to hospital transfers) were considered one event. Evidence of >1 identical physician visit-related ICD code on the same day was considered one visit.

Not counted as outcomes were pregnancy-related hospital or physician visit ICD codes (since DMD discontinuation is common during pregnancy)^20^ and neurologist specialty-specific physician visits (as their frequency is expected to be higher in persons using a DMD (versus not using), as per standard of care,^20^ and could result in the overestimation of healthcare use related to DMD prescribing).

### Statistical analyses

Data were analyzed within each province using a common approach. Aggregated results were combined using random-effects meta-analyses. We assessed the association between DMD exposure and (1) all-cause hospitalizations using a recurrent events proportional means model with robust sandwich variance estimates and (2) physician visits using a negative binomial regression model fitted by generalized estimating equations with exchangeable working correlation matrix. Findings were expressed as hazard or rate ratios, with 95% confidence intervals (CIs). These approaches allow for multiple events analyses for each participant while accounting for dependence of events. For hospitalizations, because an individual cannot be at risk of a second hospitalization while already hospitalized, the hospital stay was excluded from the follow-up period. For physician visits, the number were counted yearly, or by periods of DMD exposure, if changes in DMD use occurred within a 1-year period; an offset (log(days within each time period)) was specified to account for the variable length of periods. All models were adjusted for sex and socioeconomic status at the index date. Age, Charlson comorbidity score, and calendar-year were adjusted for as time-dependent covariates, updated on a yearly basis (the latter adjustment was made to account for any secular changes in healthcare delivery). The “DMD-unexposed” period formed the reference category for the main analyses, although the pairwise comparisons between DMDs (by generation and individual DMD) were reported from the same models as an exploratory approach. Crude rates per 100 person-years of follow-up were described.

Complementary sex and physician specialty-specific analyses were performed. The latter was applied to the physician visits only, with crude rates by person-years of follow-up described for each DMD, and during periods of no exposure. In the largest province only, British Columbia, neurologist specialty was included in the count of all physician visits as a complementary approach. SAS V.9.4 and R V.4.0.2 were used for analyses.

## Results

### Cohort characteristics

A total of 35,894 MS cases were identified; most were female (72%) and the mean age at index date was 44.6 (standard deviation (SD) = 13.6) years (Table 1). Cohort characteristics, including by DMD exposure, are published in detail.^2^ Approximately 30% of our study population were exposed to a DMD during follow-up, and this estimate falls within the ranges reported in other population-based observational studies over a comparable time period.^2^ The total person-years of follow-up while exposed to any DMD was 63,290 (10,418 individuals); any first-generation DMD was 54,605 (9204 individuals) and any second-generation DMD was 8685 (3668 individuals).

**Table 1.** Characteristics of the multiple sclerosis study population, Canada (1996–2017/2018).

Characteristics	British Columbia, *n* = 19,360	Manitoba, *n* = 5825	Nova Scotia, *n* = 5352	Saskatchewan, *n* = 5357	Overall cohort, *n* = 35,894
Sex, *n* (%)					
Female	13,940 (72.0)	4131 (70.9)	3989 (74.5)	3717 (69.4)	25,777 (71.8)
Male	5420 (28.0)	1694 (29.1)	1363 (25.5)	1640 (30.6)	10,117 (28.2)
Age at index date in years, mean (SD)	44.5 (13.5)	44.4 (13.7)	44.5 (13.2)	45.3 (14.1)	44.6 (13.6)
Socioeconomic status^a^, *n* (%)					
1 (lowest income quintile)	3763 (19.4)	885 (15.2)	909 (17.0)	872 (16.3)	6429 (17.9)
2	3695 (19.1)	1088 (18.7)	1030 (19.2)	1103 (20.6)	6916 (19.3)
3	3931 (20.3)	1255 (21.5)	1043 (19.5)	1004 (18.7)	7233 (20.2)
4	4094 (21.1)	1102 (18.9)	1034 (19.3)	1154 (21.5)	7384 (20.6)
5 (highest income quintile)	3779 (19.5)	1310 (22.5)	1014 (18.9)	988 (18.4)	7091 (19.8)
Unavailable	98 (0.5)	185 (3.2)	322 (6.0)	236 (4.4)	841 (2.3)
Comorbidity score^b^, *n* (%)					
0	15,002 (77.5)	4525 (77.7)	4149 (77.5)	4297 (80.2)	27,973 (77.9)
1	2965 (15.3)	963 (16.5)	850 (15.9)	796 (14.9)	5574 (15.5)
2	903 (4.7)	235 (4.0)	215 (4.0)	174 (3.2)	1527 (4.3)
⩾3	490 (2.5)	102 (1.8)	138 (2.6)	90 (1.7)	820 (2.3)
Calendar year at index date, *n* (%)					
1996–1999	8533 (44.1)	3120 (53.6)	2648 (49.5)	2197 (41.0)	16,498 (46.0)
2000–2004	3256 (16.8)	866 (14.9)	803 (15.0)	1066 (19.9)	5991 (16.7)
2005–2009	3161 (16.3)	724 (12.4)	802 (15.0)	873 (16.3)	5560 (15.5)
2010–2014	2942 (15.2)	767 (13.2)	765 (14.3)	772 (14.4)	5246 (14.6)
2015–2017/2018	1468 (7.6)	348 (6.0)	334 (6.2)	449 (8.4)	2599 (7.2)
Person-years of follow-up—total	225,877	75,834	68,035	61,552	431,299
Person-years of follow-up during periods of exposure to					
Any DMDs	24,970	11,890	14,191	12,239	63,290
Any first-generation DMDs	21,023	11,170	11,691	10,721	54,605
Beta-interferon^c^	15,326	8044	7702	5440	36,512
Glatiramer acetate	5697	3125	3989	5281	18,092
Any second-generation DMDs	3947	720	2500	1517	8685
Natalizumab	831	143	641	133	1748
Fingolimod	987	123	540	77	1726
Dimethyl fumarate	1246	326	795	980	3348
Teriflunomide	657	125	485	270	1538
Alemtuzumab	223	3	34	58	317
Daclizumab	<6	0	0	0	<6
Ocrelizumab	<6	0	<6	0	<6
No DMD	200,907	63,945	53,844	49,313	368,009
Number of individuals ever exposed, by type of DMD, during follow-up^d^, *n* (%)					
Any DMDs	4732 (24.4)	1762 (30.2)	2036 (38.0)	1888 (35.2)	10,418 (29.0)
First-generation DMDs –any^e^	4124 (21.3)	1694 (29.1)	1763 (32.9)	1623 (30.3)	9204 (25.6)
Beta-interferon^c^	3140 (16.2)	1294 (22.2)	1300 (24.3)	1019 (19.0)	6753 (18.8)
Glatiramer acetate	1719 (8.9)	782 (13.4)	778 (14.5)	970 (18.1)	4249 (11.8)
Second-generation DMDs–any^e^	1756 (9.1)	340 (5.8)	870 (16.3)	702 (13.1)	3668 (10.2)
Natalizumab	286 (1.5)	52 (0.9)	207 (3.9)	49 (0.9)	594 (1.7)
Fingolimod	421 (2.2)	69 (1.2)	201 (3.8)	42 (0.8)	733 (2.0)
Dimethyl fumarate	758 (3.9)	193 (3.3)	360 (6.7)	518 (9.7)	1829 (5.1)
Teriflunomide	520 (2.7)	86 (1.5)	260 (4.9)	194 (3.6)	1060 (3.0)
Alemtuzumab	179 (0.9)	6 (0.1)	39 (0.7)	71 (1.3)	295 (0.8)
Daclizumab	6 (<0.1)	0	0	0	6 (<0.1)
Ocrelizumab	<6 (<0.1)	0	7 (0.1)	0	7 (<0.1)^f^

DMD: disease-modifying drugs; SD: standard deviation.

The index date was the earlier of the first MS-specific or related demyelinating disease code recorded in any of the physician and/or hospital data or first MS DMD prescription filled; 1 January 1996 (British Columbia) or 1 April 1996 (Manitoba) or 1 January 1997 (Saskatchewan) or 1 January 1998 (Nova Scotia; the date when prescription data became first available in the respective province); a person’s 18th birthday.

As per data privacy and access agreements, small cell sizes (<6 individuals within any group) are suppressed.

a Socioeconomic status is represented by neighborhood income quintiles, as measured closest to the index date.

b Comorbidity is measured using the Charlson Comorbidity Index (modified to exclude hemiplegia/paraplegia) in the 1-year period before the index date.

c All beta-interferon products were considered as one class.

d Follow-up was from index date until the earliest of death, emigration from the province or 31 December 2017 (British Columbia, Manitoba, Nova Scotia) or 31 March 2018 (Saskatchewan) (study end date).

e Some people were exposed to >1 DMD; hence, the sum of the individual first- or second-generation DMDs exceeds the sum of any first- or second-generation DMD.

f Small cell sizes (<6 individuals) are suppressed and were not included in the total count as per the data privacy and access agreements.

### Hospitalizations

**Figure 1.**
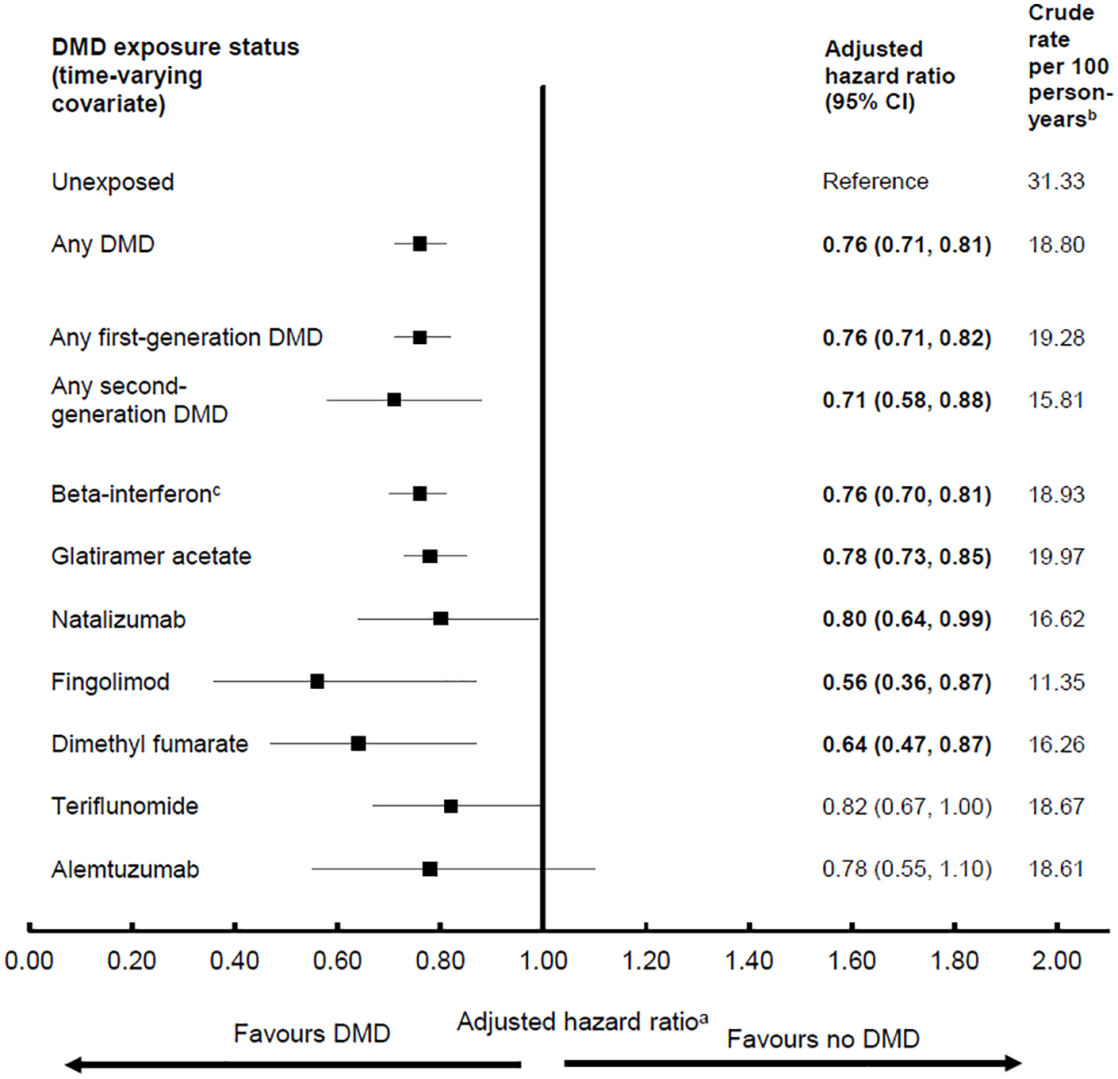
Exposure to disease-modifying drugs for multiple sclerosis and hazard of hospitalization in a population-based cohort. CI: confidence interval; DMD: disease-modifying drug. Bold indicates *p* < 0.05. ^a^Results from each of the four provinces were adjusted for sex, socioeconomic status (quintiles) closest to the index date, and the following characteristics over time: age (continuous), calendar year (continuous), and comorbidity score (categorized as 0, 1, 2, ⩾ 3) measured using a modified Charlson Comorbidity Index and were then combined using random-effects meta-analyses. ^b^Person-years of follow-up for the calculation of crude rate were as per Table 1 except the duration of a hospitalization was discounted from the follow-up time to avoid immortal time bias. ^c^All beta-interferon products were considered as one class.

Exposure to any (versus no) DMD was associated with a 24% lower hazard of hospitalization (adjusted hazard ratio, aHR: 0.76; 95% confidence interval (CI): 0.71–0.81). Similarly, for the first-generation DMDs, a 24% lower hazard was observed, with a 29% lower hazard for the second generation (Figure 1). Variation was seen across the individual DMDs, ranging from an 18% lower hazard for teriflunomide, 20% for natalizumab, 22% for glatiramer acetate and alemtuzumab, 24% for beta-interferon, to 36% for dimethyl fumarate and 44% for fingolimod. However, the HRs did not reach significance for teriflunomide and alemtuzumab (95% CIs included one).

### Physician visits

**Figure 2.**
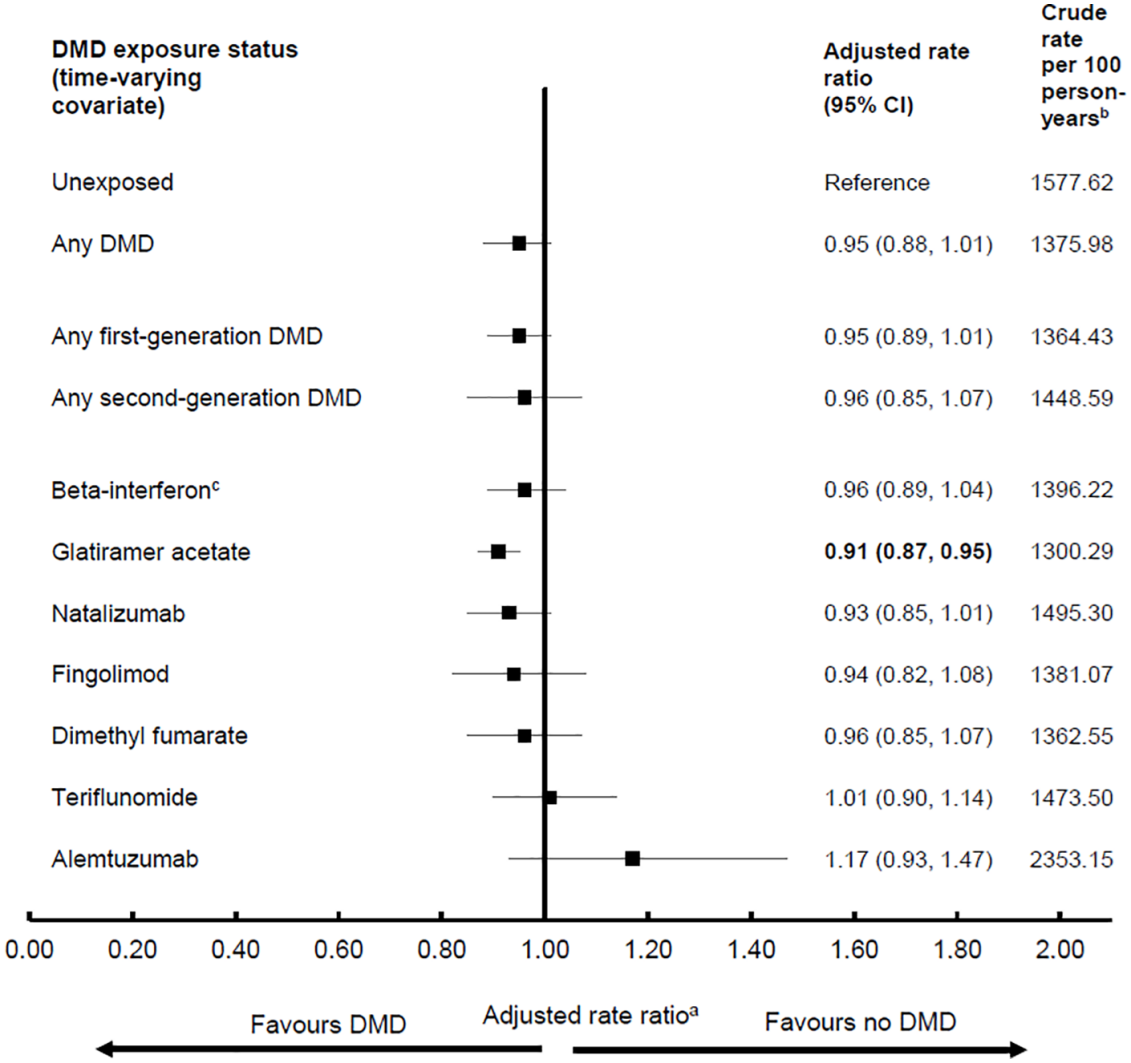
Exposure to disease-modifying drugs for multiple sclerosis and rates of physician service use. CI: confidence interval; DMD: disease-modifying drug. Bold indicates *p* < 0.05. ^a^Results from each of the four provinces were adjusted for sex, socioeconomic status (quintiles) closest to the index date, and the following characteristics over time: age (continuous), calendar year (continuous), and comorbidity score (categorized as 0, 1, 2, ⩾3) measured using a modified Charlson Comorbidity Index and were then combined using random-effects meta-analyses. ^b^Person-years of follow-up/exposure are shown in Table 1 and were used to calculate the crude rates. ^c^All beta-interferon products were considered as one class.

Overall, while exposure to any (versus no) DMD was associated with a lower rate of physician visits, this was modest and did not reach significance (adjusted rate ratio, aRR: 0.95; 95% CI: 0.88–1.01). A similar pattern was observed for exposure to a first- or second-generation DMD and for most of the individual DMDs (Figure 2). Only glatiramer acetate was associated with a significantly lower rate of physician visits (by 9%).

### Comparisons between DMDs

When the DMDs were directly compared to each other, for hospitalizations, there was no significant difference between second- and first-generation DMDs (aHR: 0.87; 95% CI: 0.66–1.17). When assessed by individual DMD, no differences were evident when the first-generation (beta-interferon or glatiramer acetate) were each compared to each of the other DMDs (Table 2). For the second-generation DMDs, natalizumab was associated with a 30%–49% higher hazard of hospitalization versus dimethyl fumarate and fingolimod.

**Table 2.** Disease-modifying drugs for multiple sclerosis: exposure by DMD and hazard of hospitalization (pairwise comparisons).

DMD exposure status (time-varying covariate)	Reference category						
	Beta-interferon	Glatiramer acetate	Natalizumab	Fingolimod	Dimethyl fumarate	Teriflunomide	Alemtuzumab
	Adjusted hazard ratio (95% CI)^a^						
Beta-interferon^b^	–	0.96 (0.90–1.03)	0.97 (0.79–1.18)	1.35 (0.83–2.20)	1.18 (0.88–1.59)	0.91 (0.71–1.18)	1.01 (0.72–1.43)
Glatiramer acetate	1.04 (0.97–1.12)	–	1.02 (0.86–1.21)	1.41 (0.89–2.23)	1.23 (0.90–1.66)	0.95 (0.72–1.24)	1.07 (0.76–1.52)
Natalizumab	1.03 (0.85–1.26)	0.98 (0.82–1.16)	–	**1.49** (1.13–1.94)	**1.30** (1.05–1.60)	1.08 (0.85–1.37)	1.14 (0.74–1.74)
Fingolimod	0.74 (0.45–1.20)	0.71 (0.45–1.12)	**0.67** (0.51–0.88)	–	0.90 (0.72–1.12)	0.71 (0.51–1.01)	0.75 (0.50–1.13)
Dimethyl fumarate	0.85 (0.63–1.14)	0.82 (0.60–1.11)	**0.77** (0.62–0.95)	1.11 (0.89–1.39)	–	0.80 (0.59–1.08)	0.96 (0.62–1.47)
Teriflunomide	1.09 (0.85–1.42)	1.05 (0.80–1.38)	0.93 (0.73–1.18)	1.40 (0.99–1.98)	1.25 (0.93–1.69)	–	1.05 (0.63–1.75)
Alemtuzumab	0.99 (0.70–1.39)	0.93 (0.66–1.32)	0.88 (0.57–1.35)	1.33 (0.89–2.00)	1.05 (0.68–1.61)	0.96 (0.57–1.60)	–

CI: confidence interval; DMD: disease-modifying drug. Bold indicates *p* < 0.05.

a Results from each of the four provinces were adjusted for sex, socioeconomic status (quintiles) closest to the index date, and the following characteristics over time: age (continuous), calendar year (continuous), and comorbidity score measured using a modified Charlson Comorbidity Index (categorized as 0, 1, 2, ⩾3) and were then combined using random-effects meta-analyses.

b All beta-interferon products were considered as one class.

For physician visits, while there was no significant difference for the second- versus first-generation DMDs (aRR: 1.01; 95% CI: 0.94–1.08), rates differed by individual DMD (Table 3). For example, alemtuzumab was associated with a 19%–34% significantly higher rate of physician visits when compared to each of the other DMDs, while teriflunomide was associated with a 10%–12% higher rate when compared to glatiramer acetate, natalizumab, and fingolimod.

**Table 3.** Disease-modifying drugs for multiple sclerosis: exposure by DMD and rates of physician service use (pairwise comparisons).

DMD exposure status (time-varying covariate)	Reference category						
	Beta-interferon	Glatiramer acetate	Natalizumab	Fingolimod	Dimethyl fumarate	Teriflunomide	Alemtuzumab
	Adjusted rate ratio (95% CI)^a^						
Beta-interferon^b^	–	1.06 (1.00–1.13)	1.08 (1.00–1.17)	1.03 (0.89–1.20)	1.01 (0.93–1.09)	0.95 (0.87–1.04)	**0.82** (0.74–0.91)
Glatiramer acetate	0.94 (0.89–1.00)	–	0.98 (0.91–1.06)	0.97 (0.87–1.08)	0.95 (0.87–1.04)	**0.90** (0.83–0.96)	**0.77** (0.64–0.94)
Natalizumab	0.92 (0.86–1.00)	1.02 (0.95–1.10)	–	0.99 (0.88–1.12)	0.95 (0.88–1.03)	**0.91** (0.83–0.99)	**0.75** (0.66–0.85)
Fingolimod	0.97 (0.83–1.12)	1.03 (0.93–1.15)	1.01 (0.90–1.14)	–	0.95 (0.88–1.02)	**0.90** (0.84–0.97)	**0.77** (0.64–0.92)
Dimethyl fumarate	0.99 (0.92–1.07)	1.05 (0.96–1.15)	1.05 (0.97–1.14)	1.06 (0.98–1.14)	–	0.95 (0.90–1.00)	**0.80** (0.70–0.91)
Teriflunomide	1.05 (0.96–1.14)	**1.12** (1.04–1.20)	**1.10** (1.01–1.20)	**1.11** (1.03–1.19)	1.06 (1.00–1.11)	–	**0.84** (0.73–0.96)
Alemtuzumab	**1.22** (1.10–1.35)	**1.29** (1.07–1.57)	**1.34** (1.17–1.52)	**1.30** (1.08–1.57)	**1.25** (1.10–1.43)	**1.19** (1.04–1.37)	–

CI: confidence interval; DMD: disease-modifying drug. Bold indicates *p* < 0.05.

a Results from each of the four provinces were adjusted for sex, socioeconomic status (quintiles) closest to the index date, and the following characteristics over time: age (continuous), calendar year (continuous), and comorbidity score measured using a modified Charlson Comorbidity Index (categorized as 0, 1, 2, ⩾3), and were then combined using random-effects meta-analyses.

b All beta-interferon products were considered as one class.

### Complementary analyses

**Figure 3.**
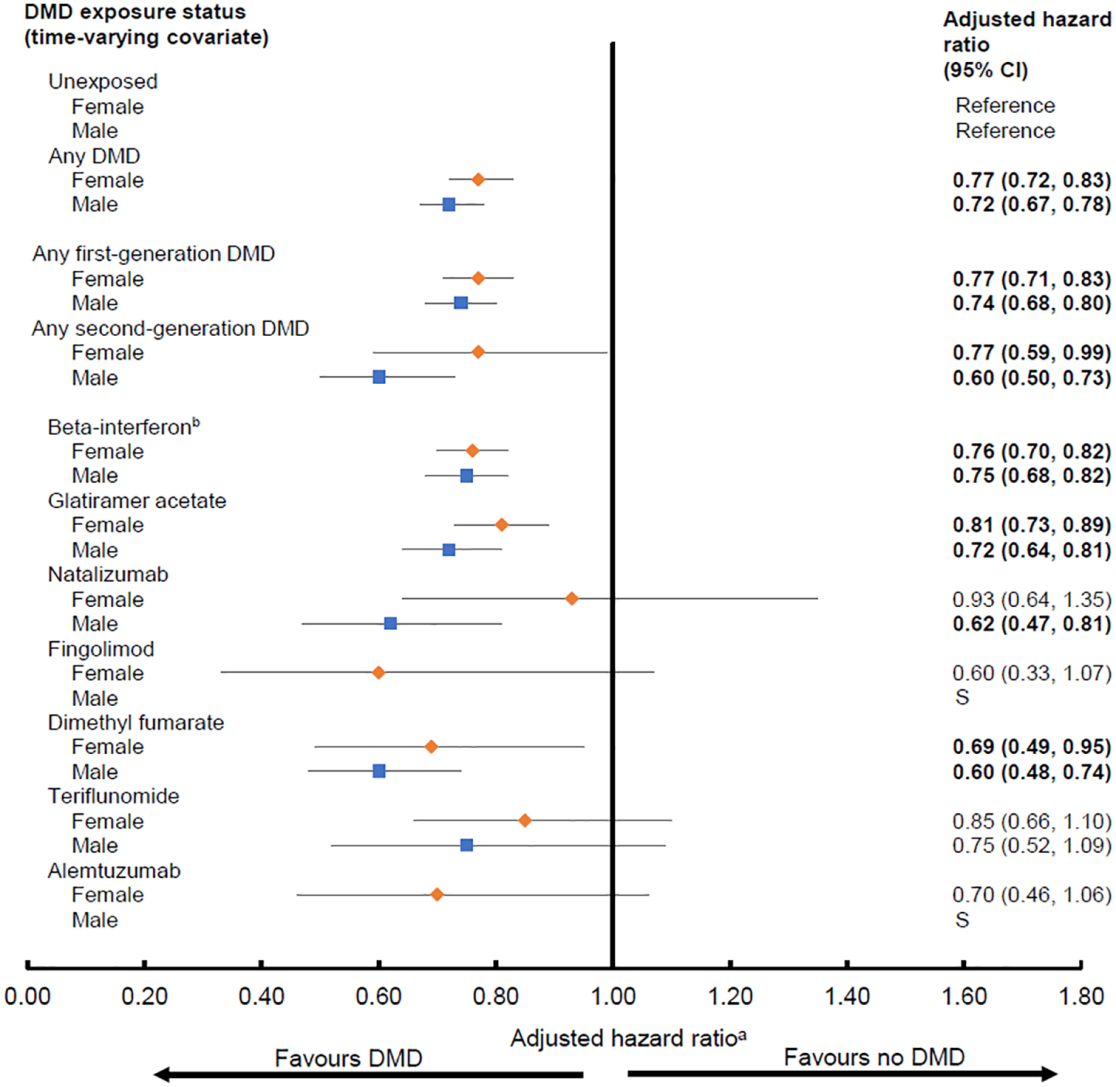
Exposure to disease-modifying drugs for multiple sclerosis and hazard of hospitalization by sex. CI: confidence interval; DMD: disease-modifying drug; S: small event rates prevented generation of reliable estimates. Bold indicates *p* < 0.05. ^a^Results from each of the four provinces were adjusted for socioeconomic status (quintiles) closest to the index date and the following characteristics over time: age (continuous), calendar year (continuous), and comorbidity score (categorized as 0, 1, 2, ⩾3) measured using a modified Charlson Comorbidity Index and were then combined using random-effects meta-analyses. Hazard ratios were estimated by introducing interaction terms between sex and DMD exposure variables. ^b^All beta-interferon products were considered as one class.

**Figure 4.**
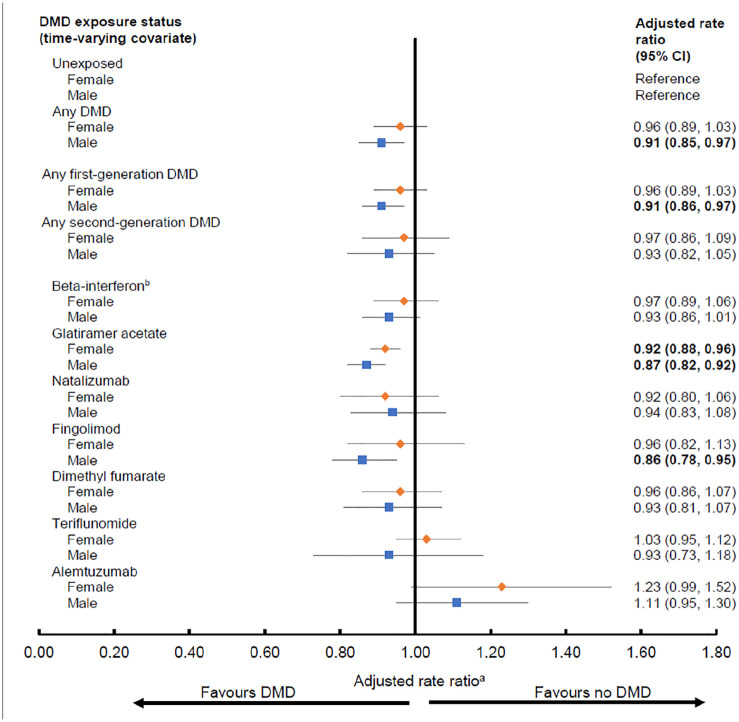
Exposure to disease-modifying drugs for multiple sclerosis and rates of physician service use by sex. CI, confidence interval; DMD, disease-modifying drug. Bold indicates *p* < 0.05. ^a^Results from each of the four provinces were adjusted for socioeconomic status (quintiles) closest to the index date and the following characteristics over time: age (continuous), calendar year (continuous), and comorbidity score (categorized as 0, 1, 2, ⩾3) measured using a modified Charlson Comorbidity Index and were then combined using random-effects meta-analyses. Rate ratios were estimated by introducing interaction terms between sex and DMD exposure variables. ^b^All beta-interferon products were considered as one class.

The direction of findings was generally similar for both sexes, although some estimates were more pronounced (lower) for males (Figures 3 and 4). For hospitalizations, this was most evident for the second-generation DMDs; in particular natalizumab was associated with a lower hazard for males (aHR: 0.62; 95% CI: 0.47–0.81), but not for females (aHR: 0.93; 95% CI: 0.64–1.35, Figure 3).

**Figure 5.**
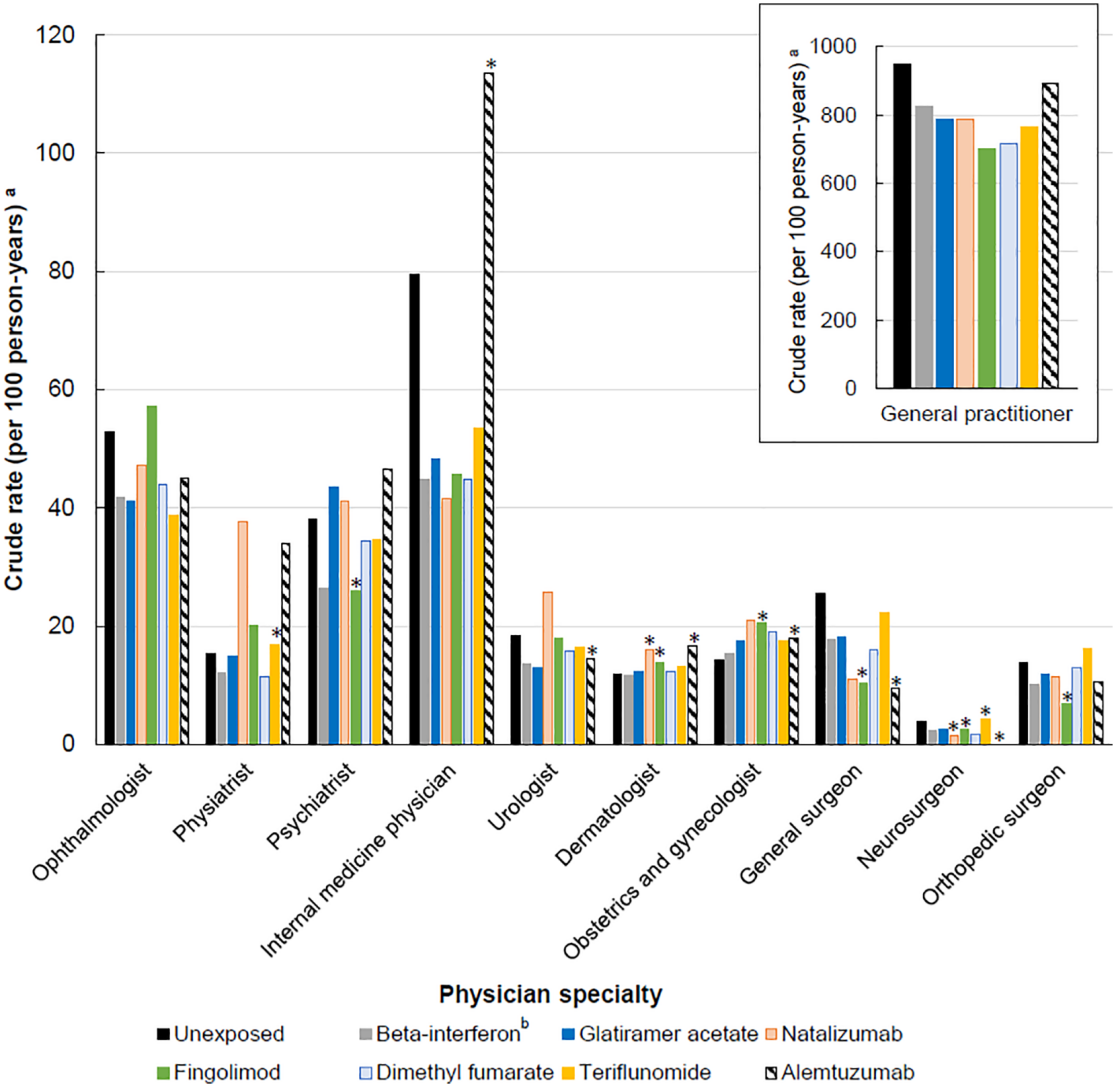
Exposure to disease-modifying drugs for multiple sclerosis and rates of physician visits by physician specialties. ^a^Results from four provinces were combined and the crude rate was calculated using the formula: (total number of physician claims for each specialty by DMD exposure status/total person years of follow-up by DMD exposure status) × 100. The results for general practitioner were shown in the right square box with a separate *y*-axis value (i.e. with a 10 times higher *y*-axis value than the remaining physician specialties). ^b^All beta-interferon products were considered as one class. *As per the data privacy and access agreements, small cell size (<6 total number of physician claims reported in one or more provinces) is suppressed and were not included in the total count for that estimation of crude rate.

Crude rates of physician visits varied by individual DMD and relative to periods of no exposure (Figure 5). Fingolimod was associated with relatively high rates of ophthalmologist visits (57.17/100 person-years), while alemtuzumab with general practitioner (893.94/100 person-years), internal medicine (113.44/100 person-years), psychiatry (46.63/100 person-years), and physiatry visits (34.02/100 person-years). Periods of no exposure were associated with relatively high rates of general practitioner (949.70/100 person-years) and internal medicine visits (79.54/100 person-years) and three surgical specialty visits (general, neuro-, and orthopedic). When we included neurologist specialty in the overall number of physician visits the findings were consistent with those from the main analysis (using data from British Columbia only; Supplementary Figure 1).

## Discussion

We examined the relationship between DMD exposure and health service use in a large population of individuals with MS in a universal healthcare setting, spanning >20 years. Exposure to any first-generation DMD was associated with a 24% lower hazard of hospitalization compared to no exposure, rising to 29% for the second-generation DMDs. Variation was seen across the individual DMDs, but all findings were suggestive of lower hospitalizations, ranging from 18% for teriflunomide to 44% for fingolimod. In contrast, DMD exposure (whether assessed by generation or individual DMD) versus no exposure was generally not associated with substantial differences in the overall rate of physician visits. Our study provides real-world evidence of a beneficial relationship between DMD exposure and hospitalizations.

The relationship between DMD exposure and healthcare use may be explained by several factors. The DMDs have been shown to reduce the frequency and severity of relapses, which in turn could lower the risk of hospitalization.^4,5,21,22^ Conversely, the DMDs are associated with various adverse events (AEs), including rare, but severe, events which may also pose a risk for hospitalization^23^ or may require regular physician visits for management and monitoring.^24^ We found relatively few studies with which to compare our findings.^4–7,25^ For example, two studies (from Saskatchewan, Canada, and Finland) took a broad ecological approach and examined the relationship between DMD and healthcare use at the population rather than individual level.^6,7^ In Saskatchewan, as the overall number of filled DMD prescriptions increased all-cause hospitalizations decreased (1997–2016), while there was no relationship with the number of physician service claims.^6^ In Finland, a 4% annual reduction in MS-related hospitalizations (2004–2014) coincided with the increased availability and utilization of the DMDs.^7^ Which individual DMDs were contributing to these patterns was not examined in either study.^6,7^ Two US studies (funded by the same pharmaceutical company) accessed data spanning a 2-year period on select groups of people with private insurance.^4,5^ They found a lower proportion of people hospitalized in the year after (versus before) DMD initiation. Neither study could assess whether participants left the insurance plan during follow-up and data beyond 2 years were unavailable.^4,5^ A third US-based study of Medicare enrollees reported fewer all-cause hospitalizations related to DMD use; however, interpretation of findings is limited due to its cross-sectional design and 1-year study period.^25^

Interesting differences emerged when the individual DMDs were directly compared to each other as an exploratory approach. For example, of the second-generation DMDs, natalizumab was associated with higher hazard of hospitalization versus two of the oral DMDs (fingolimod and dimethyl fumarate). Our findings might reflect natalizumab’s status as a second-line treatment in Canada; it is typically reserved for patients with an inadequate response to another DMD due to its risk-benefit profile.^2^ This could mean that patients with more active disease might be exposed to natalizumab, which could result in more hospitalizations due to underlying disease activity. Some studies have compared natalizumab with fingolimod in relation to effectiveness, assessed by relapse rates and disability progression, but the findings have been mixed; few studies reported no differences,^26,27^ while others concluded that natalizumab seemed more effective than fingolimod, at least over the short term.^28,29^ Exposure to two DMDs—alemtuzumab and teriflunomide—was associated with higher rates of physician visits in our study. Rates were 19%–34% higher for alemtuzumab compared with each of the other DMDs, which likely reflects the intensive (monthly) monitoring and laboratory testing required.^30^ Whereas for teriflunomide, rates were 10%–12% higher relative to glatiramer acetate, natalizumab, and fingolimod. For example, teriflunomide requires frequent blood tests, especially during the first 6 months of treatment,^31^ while glatiramer acetate does not.^24^ Frequent laboratory testing can also result in incidental findings, unrelated to DMD, which in turn may necessitate further investigations and physician visits.^32^ Teriflunomide has been shown as less efficacious^1^ and less effective^33^ than either fingolimod or natalizumab in managing disease activity, which might also partly contribute to our observations. Thus, it is plausible that any increases in safety monitoring may be counterbalanced by a decrease in the need for physician services related to drug (in)effectiveness.

We also examined crude rates of physician services by specialty. Exposure to fingolimod was associated with relatively high rates of ophthalmologist visits, which would concur with the recommendation for regular ocular assessments and risk of macular edema.^34^ Alemtuzumab exposure was associated with high rates of general practitioner and internal medicine visits, likely reflecting the regular safety-related monitoring required and its AE profile, including risk of autoimmune disease.^30^ Why alemtuzumab was also associated with high rates of psychiatric visits was less clear. A systematic review found no significant association between any individual DMD, including alemtuzumab (based on randomized-controlled trials), and an increased risk of adverse psychiatric effects.^35^

Although our findings were generally similar for both males and females, there was some suggestion that the effects were more pronounced for males. We were unable to find another study with which to compare these sex-specific findings. DMD-related sex differences appears poorly understood, despite well-recognized sex-differences in pharmacokinetics/pharmacodynamics.^36,37^ A 2018 review found that the majority (65%) of MS clinical trials did not pre-plan a sex-specific analysis.^37^ Of those that did, only efficacy was examined (not harms) and while most found no sex differences, studies may have lacked sufficient statistical power as relatively few males were included.^36,37^ Although males comprised approximately 30% of individuals in our study, our cohort was sizable. Nonetheless, our cohort was insufficient to reliably examine sex differences for the more recent DMDs.

Our study has several limitations. While administrative health data offer opportunity to examine the patterns of health service utilization in the MS population at the individual level, these patterns should be interpreted accordingly; health services use may reflect, in part, a balance between the potential harms and benefits of the DMDs and should not be interpreted as only representing one or the other. We were not able to consider MS-specific clinical characteristics, such as relapses or disability which are not captured in the administrative data. Despite adjusting for several demographic characteristics and comorbidity, it is possible that comparisons between individual DMDs were influenced by underlying clinical features or other unmeasured confounders. For example, patients with more active disease might be treated with a higher efficacy DMD, but might also have required frequent physician visits or inpatient management due to their underlying disease. However, our complementary analyses, in which we included neurologist visits, did not change interpretation of findings. We had limited power to assess some of the newer second-generation DMDs which did not become widely available until close to our study end. Consequently, for DMDs such as alemtuzumab, the total person-years exposed was modest. We did not explore cause-specific events as, particularly for hospitalizations, modest number of events would prevent generation of reliable estimates. Other information such as lifestyle behaviors (e.g. smoking) or race/ethnicity or access to DMD as part of a clinical trial were not available. For example, aside from a very small number of individuals that may have enrolled in a clinical trial and been randomized to receive a DMD, most individuals would not have been on a DMD before the index date.^2^ Nonetheless, we were able to account for several important factors which may influence treatment decisions and outcomes, such as socioeconomic status, sex, age, comorbidity burden, and calendar-year over time. We used a time-dependent approach when examining DMD exposure, which accounts for the changing treatment status of persons over time (an important consideration in minimizing bias, for example, immortal time bias). It could be value for future studies to explore different ways of grouping the DMDs (e.g. by relative efficacy or by mechanism of action). Other study strengths included avoidance of selection bias (by accessing objectively collected population-based data) and a long follow-up duration. The universal healthcare setting allowed us to provide a comprehensive evaluation of health service utilization, including all hospital admission, physician visits, and filled prescriptions, regardless of a person’s ability to pay. Few world regions have access to such comprehensive, linked administrative health data for the entire population at the individual level.

## Conclusion

We found in our study that periods of exposure to any DMD, or any first- or second-generation DMD, were associated with a lower hazard of hospitalization relative to no DMD exposure. However, there was substantial variation across the individual DMDs, particularly for the second-generation DMDs. In contrast, the relationship between DMD exposure with overall rates of physician visits was rather modest. Our study provides real-world insights into the beneficial effects of different DMDs on hospitalizations for people living with MS.

## Supplemental Material

sj-docx-1-msj-10.1177_13524585211063403 – Supplemental material for Disease-modifying drugs for multiple sclerosis and subsequent health service use Supplemental material, sj-docx-1-msj-10.1177_13524585211063403 for Disease-modifying drugs for multiple sclerosis and subsequent health service use by Huah Shin Ng, Feng Zhu, Elaine Kingwell, Yinshan Zhao, Shenzhen Yao, Okechukwu Ekuma, Lawrence W Svenson, Charity Evans, John D Fisk, Ruth Ann Marrie and Helen Tremlett in Multiple Sclerosis Journal
