# Supplementary material for: Disease-modifying drugs for multiple sclerosis and subsequent health service use
Source: Mult Scler. 2021 Dec 24;28(4):583–96. doi: 10.1177/13524585211063403 (PMC8958569; doi:10.1177/13524585211063403)
Supplement: sj-docx-1-msj-10.1177_13524585211063403 – Supplemental material for Disease-modifying drugs for multiple sclerosis and subsequent health service use [file sj-docx-1-msj-10.1177_13524585211063403.docx]

**Supplementary Materials**

**Supplementary Table 1** Diagnostic codes for MS and other central nervous system demyelinating diseases

| **Diseases** | **ICD-9 code** | **ICD-10 code** |
| --- | --- | --- |
| Multiple sclerosis | 340 | G35 |
| Optic neuritis | 377.3 | H46 |
| Acute transverse myelitis | 323.82, 341.2 | G37.3 |
| Acute disseminated encephalomyelitis | 323 | G36.9 |
| Demyelinating disease of central nervous system (CNS) unspecified | 341.9 | G37.8 |
| Other acute disseminated demyelination | NA | G36 |
| Neuromyelitis optica | 341.0 | G36.0 |

**Supplementary Table 2** The disease-modifying drugs used to treat multiple sclerosis grouped by generation and class, along with respective Health Canada approval year (through to 2017)

| **DMD generation (first or second)** | **DMD class** | **Brand name & related details** | **Health Canada approval date** |
| --- | --- | --- | --- |
| First | Beta-interferon^a^ | Interferon beta-1b [Betaseron®] (0.3 mg/vial) | July 1995 |
|  |  | Interferon beta-1b [Extavia®] (0.3mg/vial) | November 2009 |
|  |  | Peginterferon beta-1a [Plegridy®] (125mcg/0.5ml); (94mcg/0.5ml); (63 mcg/0.5ml);  (starter pack; 63 µg/0.5ml & 94 µg/0.5ml) | August 2015 |
|  |  | Interferon beta-1a [Avonex®] (30 μG/kit); (30 μG/0.5 ml) | April 1998 |
|  |  | Interferon beta-1a [Rebif®] (initiation pack); (8.8 μG); (11 μG); (22 μG); (44 μG); (66μG); (132μG) | February 1998 |
| First | Glatiramer acetate | Copaxone® (20mg/1 vial); (20mg/1 ml); (40mg/1ml)  Glatect® (20mg/1 ml) | October 1997,  August 2017 |
| Second | Natalizumab | Tysabri® (300mg/15ml) | September 2006 |
| Second | Fingolimod | Gilenya® (0.5mg capsule) | March 2011 |
| Second | Dimethyl fumarate | Tecfidera® (120 mg capsule)  Tecfidera® (240 mg capsule) | April 2013 |
| Second | Teriflunomide | Aubagio® (14 mg tablet) | November 2013 |
| Second | Alemtuzumab | Lemtrada® (12 mg/1.2ml) | December 2013 |
| Second | Daclizumab^b,c^ | Zinbryta® (150mg/ml pre-filled syringe);  (150mg/ml pre-filled pen) | December 2016 |
| Second | Ocrelizumab^c^ | Ocrevus® (300mg/ml) | August 2017 |

^a^All beta-interferon products were considered as one class.

^b^Daclizumab was withdrawn from the market in March 2018 due to safety concerns.

^c^Neither daclizumab nor ocrelizumab were assessed as individual DMD due to insufficient exposure in our cohorts.

Cladribine was not included as it was not available at the time of study (cladribine was approved by Health Canada in November 2017, and was only become available for use in clinical practice after the study end date).

Information on experimental treatments, such as immune-ablation stem cell transplant therapies, were not assessed.

**Supplementary Figure 1.** Exposure to disease-modifying drugs for multiple sclerosis and rates of physician service use, with and without neurologist visits


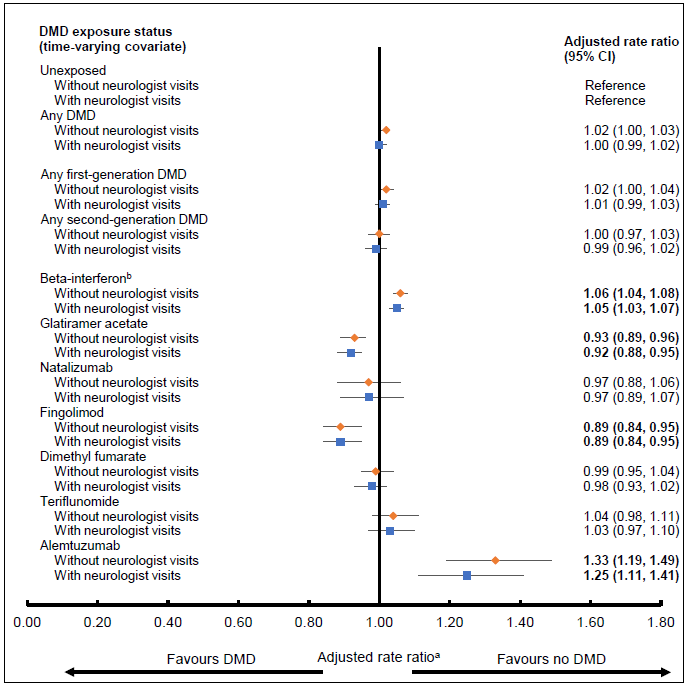


Key: CI, confidence interval; DMD, disease-modifying drug. Bold indicates p<0.05.

^a^Results shown are based on the largest province (British Columbia) and were adjusted for sex and socioeconomic status (quintiles) closest to the index date, and the following characteristics over time: age (continuous), calendar year (continuous), and comorbidity score (categorized as 0, 1, 2, ≥3) measured using a modified Charlson Comorbidity Index.

^b^All beta-interferon products were considered as one class.
